# Gastric Volvulus as a Complication of Giant Hiatal Hernia: A Case Report and Literature Review

**DOI:** 10.7759/cureus.84257

**Published:** 2025-05-16

**Authors:** Marco A Urbina Velázquez, José Emiliano González Flores, Cynthia M Portales Rivera, Michelle Cruz Méndez, Alfonso Sandoval, Frida M Silva Cruz, Hannah N Ortega Aranda, Jorge Noceda Crispin, Óscar T Teramoto Matsubara

**Affiliations:** 1 Department of Surgery, ABC Medical Center, Mexico City, MEX; 2 School of Medicine and Health Sciences, Tecnológico de Monterrey, Campus Ciudad de Mexico, Mexico City, MEX; 3 School of Medicine and Health Sciences, Tecnológico de Monterrey, Campus Ciudad de México, Mexico City, MEX; 4 Department of Investigation, National Institute of Respiratory Diseases, Instituto Nacional de Enfermedades Respiratorias (INER), Mexico City, MEX; 5 Department of Gastroenterology, ABC Medical Center, Mexico City, MEX

**Keywords:** esophagogram, gastric volvulus, giant hiatal hernia, laparoscopic surgery, organoaxial rotation

## Abstract

Gastric volvulus is a rare but potentially life-threatening condition that can arise as a complication of a giant hiatal hernia. It results from an abnormal rotation of the stomach, which can lead to obstruction, ischemia, or even gastric necrosis. Prompt recognition and surgical intervention are essential to prevent serious outcomes.

We present the case of a 58-year-old female patient with a history of hypertension who arrived at the emergency department with acute-onset severe epigastric pain (10/10 on the Visual Analog Scale), accompanied by nausea but no vomiting. Physical examination revealed involuntary guarding in the epigastric region. Initial imaging with contrast-enhanced esophagogram showed a giant hiatal hernia with intrathoracic migration of the stomach and signs of gastric volvulus. A subsequent computed tomography (CT) scan confirmed the herniation of the gastric antrum through the esophageal hiatus with organoaxial rotation.

The patient underwent urgent laparoscopic surgery. Intraoperatively, the stomach was found to be viable, with no evidence of necrosis or perforation. The herniated stomach was reduced, and the esophageal hiatus was dissected and repaired using a prosthetic mesh. The patient had an uneventful postoperative course and was discharged on the second postoperative day. She remains asymptomatic at follow-up.

This case highlights the importance of early diagnosis and intervention in patients with acute gastric volvulus, especially when associated with a giant hiatal hernia. Laparoscopic repair remains the gold standard, offering reduced morbidity, faster recovery, and favorable long-term outcomes. A literature review confirms that surgical correction of the anatomical defect is essential to prevent recurrence and severe complications such as ischemia or gastric necrosis. Endoscopic and percutaneous decompression may serve as temporary measures but are not substitutes for definitive surgical treatment.

## Introduction

The management of gastric volvulus associated with hiatal hernia involves clinical evaluation and surgical strategies based on the severity of the condition. This rare but critical disorder requires early intervention to prevent complications such as gastric ischemia or necrosis [1-4]. Laparoscopy is considered the gold standard due to its favorable outcomes [1,5]. Alternatives such as endoscopic management or percutaneous gastrostomy may be useful in selected patients but do not replace definitive correction of the anatomical defect [2,3]. We present the case of a 58-year-old female patient with no history of previous surgeries.

## Case presentation

A 58-year-old female patient with a past medical history of systemic arterial hypertension treated with telmisartan and no history of previous surgeries presented to the emergency department with sudden-onset, severe epigastric pain rated 10/10 on the Visual Analog Scale (VAS), accompanied by nausea without vomiting and no other associated symptoms. Physical examination revealed abnormal vital signs: blood pressure of 128/88 mmHg, heart rate of 111 beats per minute, respiratory rate of 22 breaths per minute, body temperature of 36.6°C, and oxygen saturation of 94% on room air. Chest auscultation revealed bilateral decreased breath sounds and tachycardia, while abdominal examination showed tenderness in the epigastric region with involuntary muscle guarding and a negative Murphy's sign.

**Figure 1:**
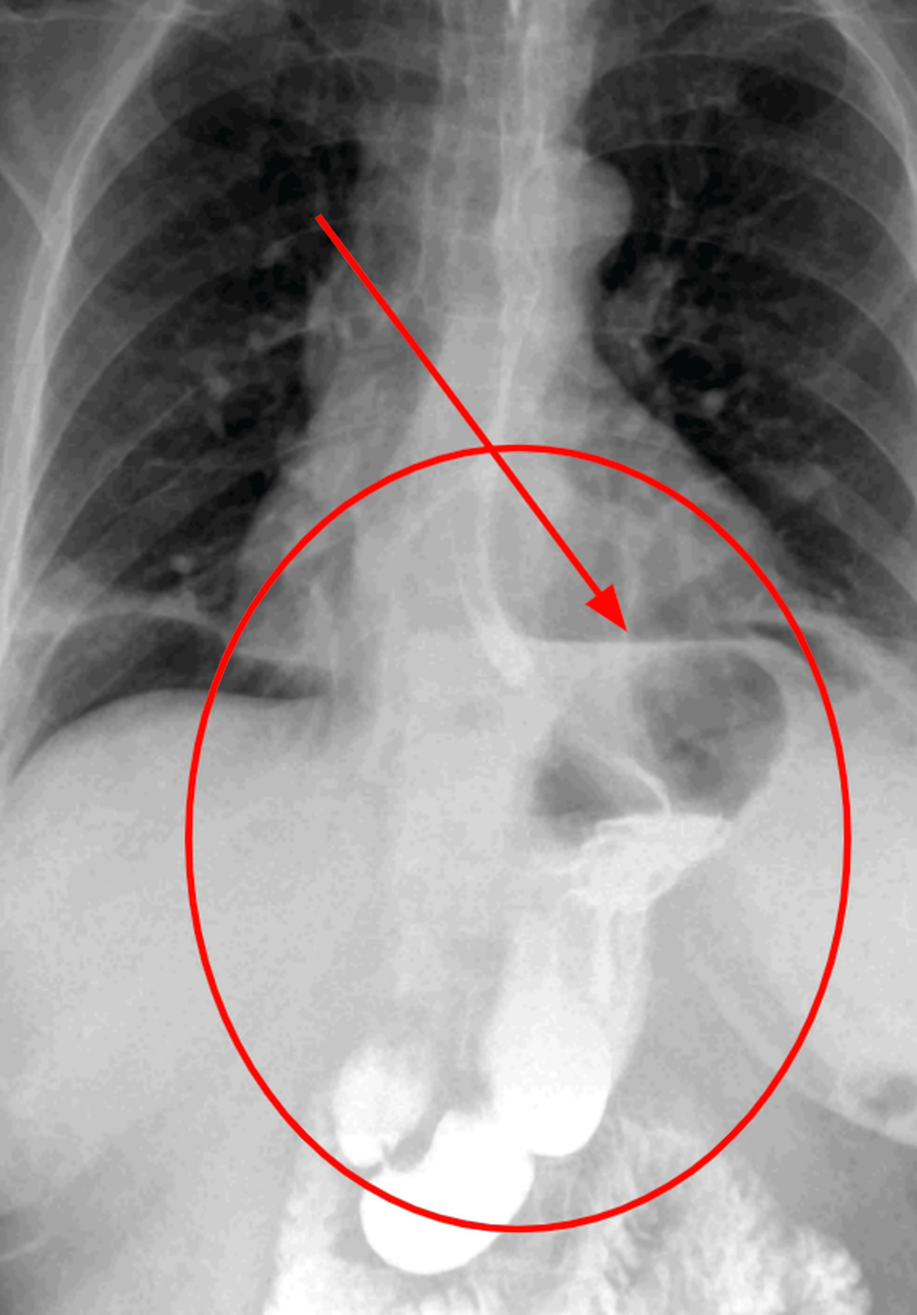
Upper gastrointestinal contrast study showing a large intrathoracic stomach. A frontal radiograph with oral contrast demonstrates a markedly distended stomach herniated into the thoracic cavity (outlined by the red circle), consistent with a giant hiatal hernia. The red arrow indicates the transition point between the esophagus and the volvulized portion of the stomach, suggesting organoaxial gastric volvulus. Air-fluid levels and abnormal gastric positioning above the diaphragm are evident, supporting the diagnosis. This imaging finding correlates with the patient's acute epigastric pain and clinical signs of gastric obstruction.

A contrast-enhanced esophagogram (Figure 1) showed upward displacement of the gastric fundus and body into the thoracic cavity, suggestive of a giant hiatal hernia. The stomach appeared twisted in a double-bubble or double-chamber configuration, indicating intrathoracic gastric volvulus. The gastroesophageal junction also appeared displaced into the thorax. Partial obstruction was noted with contrast flow.

**Figure 2:**
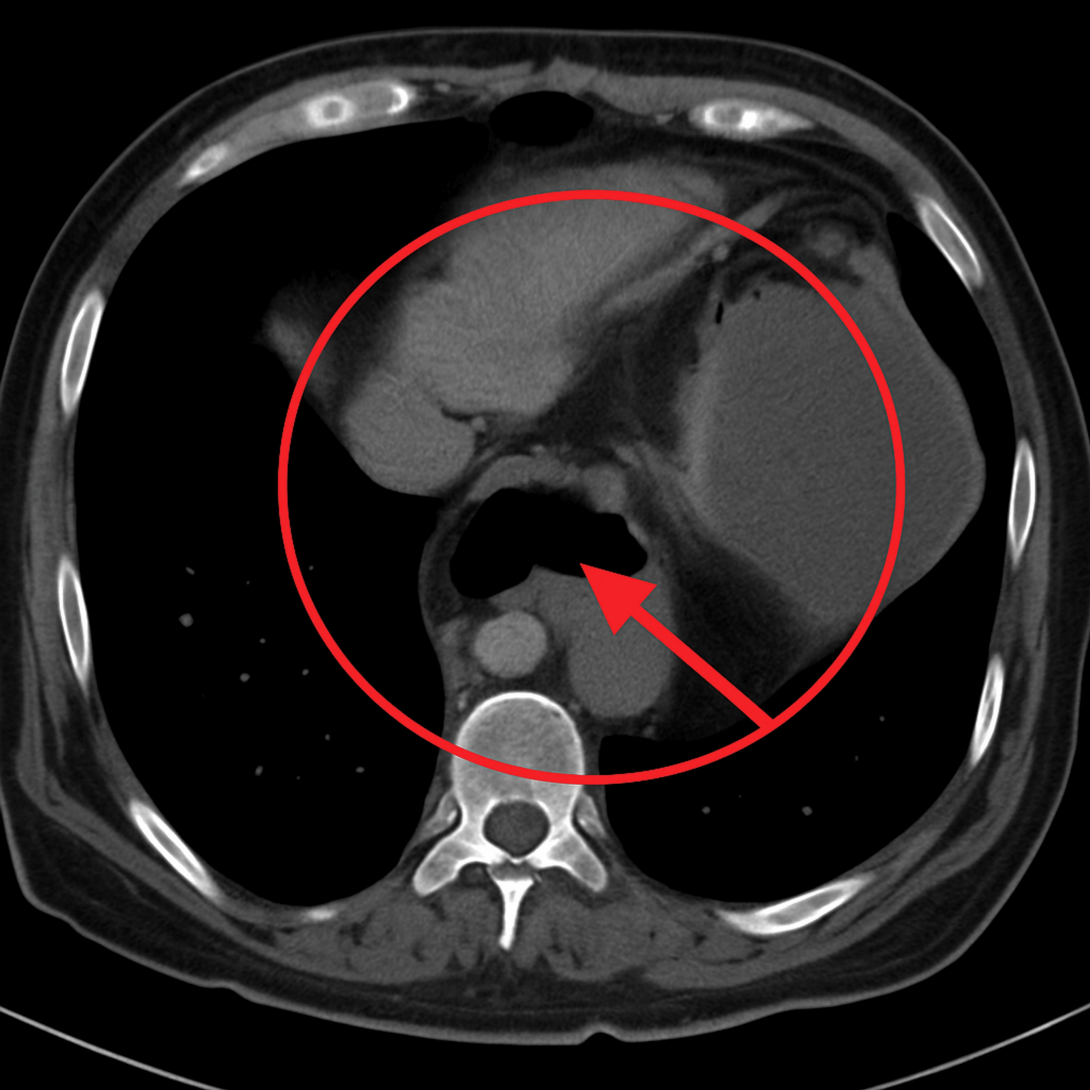
Axial contrast-enhanced computed tomography image at the level of the lower thorax demonstrates a diaphragmatic defect at the esophageal hiatus (red arrow), with intrathoracic herniation of the gastric fundus and body. The stomach appears dilated and rotated within the posterior mediastinum, consistent with organoaxial gastric volvulus associated with a giant hiatal hernia.

Subsequently, a non-contrast multidetector computed tomography (MDCT) scan was performed (Figure 2), followed by oral contrast administration. It demonstrated the gastroesophageal junction in an infradiaphragmatic position, with herniation of the gastric antrum and omental fat through the esophageal hiatus. Laboratory test results are summarized in Table 1.

**Table 1:** Initial laboratory parameters on admission compared to standard reference ranges in adult.

Parameter	Patient's value	Normal reference range
Hemoglobin	13.3 g/dL	12-16 g/dL
White blood cells	11.2×10³/μL	4.5-11.0×10³/μL
Platelets	254,000×10³/μL	150,000-450,000×10³/μL
Glucose	128 mg/dL	70-99 mg/dL (fasting)
Creatinine	1.2 mg/dL	0.6-1.1 mg/dL
Sodium	132 mEq/L	135-145 mEq/L
Potassium	3.7 mEq/L	3.5-5.0 mEq/L
Chloride	98 mEq/L	98-106 mEq/L

**Figure 3:**
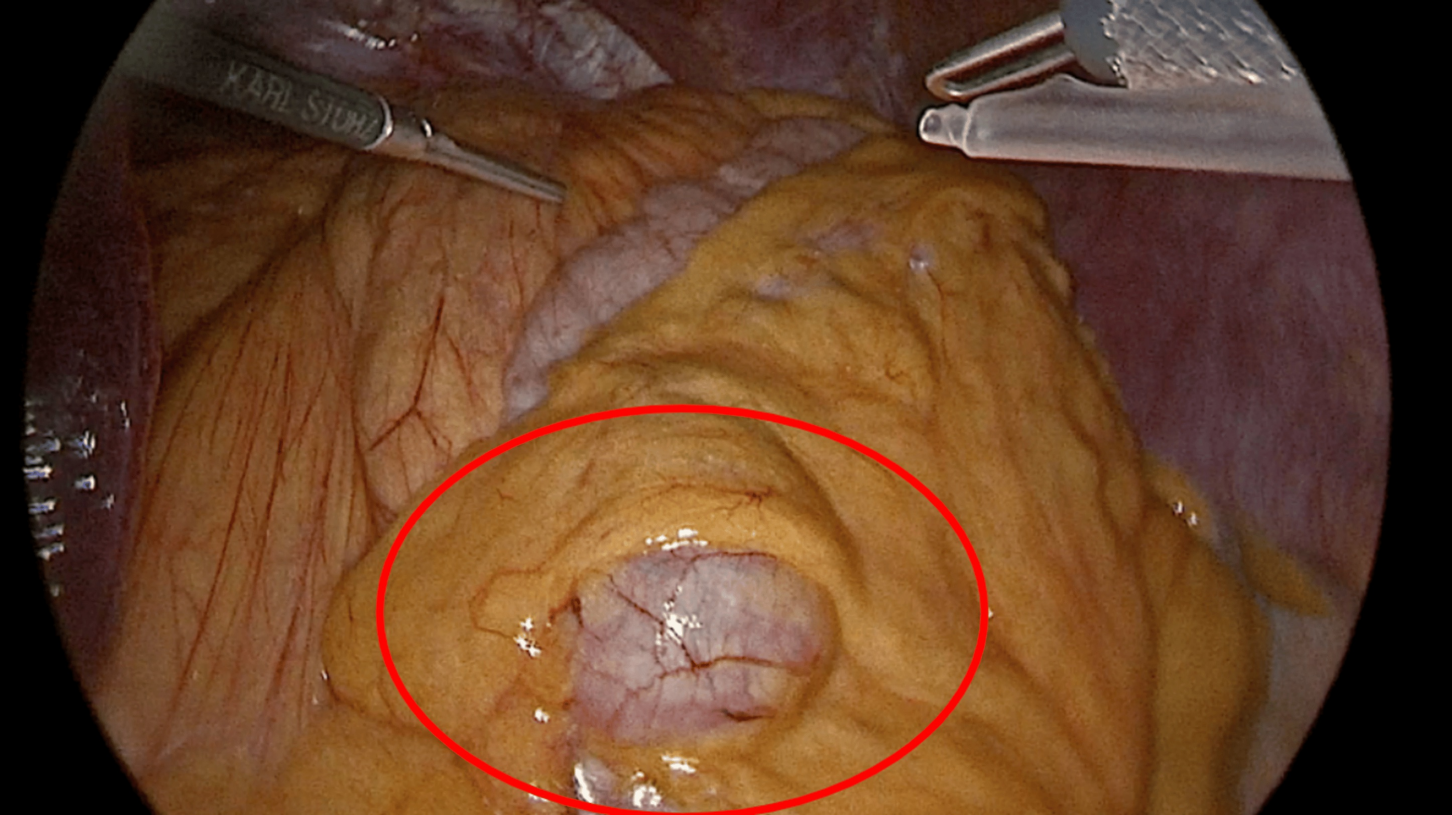
Laparoscopic intraoperative view of the herniated gastric fundus partially enveloped by the greater omentum. The fundus, indicated by the red circle, appears engorged and displaced into the mediastinum through the diaphragmatic defect. This finding is consistent with a giant hiatal hernia complicated by organoaxial volvulus. The instruments are used for the gentle dissection and mobilization of the herniated structures.

**Figure 4:**
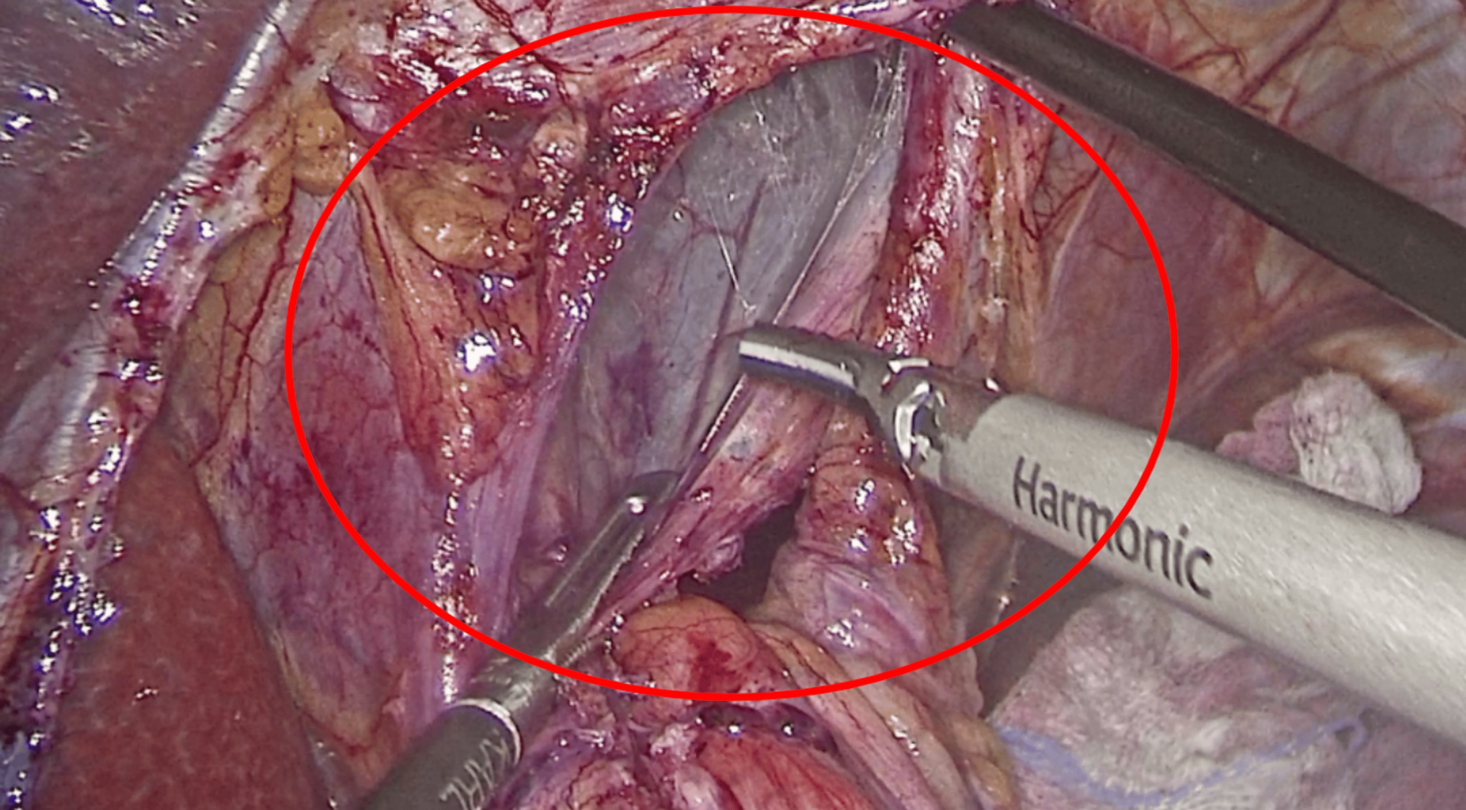
Intraoperative laparoscopic view showing posterior cruroplasty. The crural fibers are clearly visualized and are being approximated with a nonabsorbable suture using extracorporeal knot tying technique. The red circle highlights the repaired diaphragmatic hiatus following reduction of the herniated stomach, as part of the hiatal hernia repair.

Intraoperative findings were documented sequentially as the laparoscopic procedure progressed. The patient underwent surgical intervention within the first few hours of arrival at the emergency department, following prompt clinical and radiological assessment. Initially, the greater omentum was retracted, allowing for the adequate visualization of the gastric fundus, which showed preserved vascularization (Figure 3). Subsequently, a portion of the stomach was identified herniating through the esophageal hiatus, with no evidence of necrosis, perforation, or free intraperitoneal content, findings consistent with preserved gastric viability. After complete reduction of the stomach into the abdominal cavity, dissection of the esophageal hiatus was performed, achieving full exposure of the abdominal esophagus. A prosthetic mesh was then placed to reinforce the repair of the hiatal defect (Figure 4).

On postoperative day 1, a contrast esophagogram showed no signs of leakage, and oral intake was initiated with liquids, advancing to a soft diet. By postoperative day 2, the patient reported adequate pain control and was discharged on a soft diet. The patient was followed up clinically for three months after surgery. No further imaging was deemed necessary as the patient remained asymptomatic, with no signs of gastrointestinal symptoms, recurrence, or complications. The absence of clinical signs of obstruction, reflux, or pain supported the successful outcome of the procedure.

## Discussion

Our findings are consistent with the current literature, which emphasizes the importance of early diagnosis and prompt surgical intervention to prevent severe complications such as ischemia and perforation. As reported by Teague et al. and Milne et al. [6,7], laparoscopic repair remains the standard approach due to its association with lower morbidity and shorter recovery time. In our case, the early laparoscopic intervention allowed for the complete reduction of the volvulus, evaluation of gastric viability, and definitive repair with mesh reinforcement, strategies aligned with best practices described in recent case series and reviews. Additionally, the absence of necrosis or perforation in our patient, along with a favorable postoperative course and no recurrence at follow-up, supports the positive outcomes consistently reported in the literature for patients managed surgically within the early stages of gastric volvulus.

In patients with acute manifestations, such as severe epigastric pain or vomiting, surgical treatment is the main therapeutic strategy. The laparoscopic technique has become the gold standard in most cases due to its lower morbidity, shorter hospital stays, and faster postoperative recovery compared to open surgery [1,2]. This approach allows for the anatomical correction of the volvulus, reduction of the hernia, and implementation of complementary procedures, such as fundoplication, to minimize the risk of gastroesophageal reflux and clinical recurrence [3,4].

Endoscopic management may serve as an initial measure for gastric decompression and symptomatic relief in selected patients. However, this approach does not resolve the underlying anatomical defect and is therefore not considered a definitive solution [1,5]. In patients with high surgical risk due to significant comorbidities, percutaneous gastrostomy offers an alternative for continuous gastric decompression. Nonetheless, it carries potential complications such as local infection, gastric leakage, or tube displacement [8].

In severe cases involving gastric necrosis, surgical resection, either partial or total gastrectomy, is imperative. These interventions, however, carry high mortality rates, nearing 30% in most reported series, largely due to the critical condition of the patients at the time of intervention [9]. Therefore, early surgical management is crucial to avoid progression to these critical complications.

Surgical repair of the hiatal hernia is an essential component of long-term treatment. Primary hiatal closure is commonly used; however, in larger defects, prosthetic mesh reinforcement may be necessary. Although mesh reduces recurrence rates, it carries specific risks such as erosion or migration [10]. Despite these concerns, long-term outcomes with laparoscopic repair are encouraging, with significantly reduced recurrence rates when combined with fundoplication and proper anatomical correction of the hiatus [2,3].

Although laparoscopic repair is widely regarded as the gold standard for managing gastric volvulus associated with hiatal hernia, not all cases of gastrointestinal obstruction follow a predictable pattern or allow for standard approaches. In complex or atypical scenarios, such as patients with previous undiagnosed congenital anomalies, adhesions, or incidental intraoperative findings, surgeons may need to adapt their strategy. For example, Koenig and Turner described a case of gallstone ileus that was unexpectedly managed through resection of an incidentally discovered Meckel's diverticulum, underscoring the value of intraoperative flexibility and real-time clinical judgment in managing acute abdominal conditions [11]. These situations highlight that, beyond standard algorithms, surgical success may depend on the ability to individualize the approach based on findings encountered during exploration. In our case, early laparoscopic intervention allowed for the prompt reduction of the volvulus and hernia while preserving gastric viability and avoiding more radical procedures, such as gastrectomy, which are often necessary in delayed or complicated presentations.

## Conclusions

Gastric volvulus associated with hiatal hernia, though uncommon, is a complex condition that requires early intervention to avoid severe complications such as ischemia or perforation. Laparoscopy remains the gold standard due to its favorable outcomes in terms of morbidity, hospitalization, and recovery. While endoscopic management or percutaneous gastrostomy may be useful in selected patients, they do not replace definitive anatomical correction. Surgical repair, combined with techniques such as fundoplication, is essential to ensure long-term success and prevent recurrence. The definitive treatment goals can be summarized as reduction of the volvulus, prevention of recurrence, and correction of predisposing factors. The therapeutic pillars of this pathology include complete reduction of the volvulus and hernia, evaluation of gastric viability with consideration of gastrectomy, diaphragmatic repair with or without mesh placement, and securing the stomach in the subdiaphragmatic position through fundoplication or gastropexy.
